# ACL Reconstruction: Which Additional Physiotherapy Interventions Improve Early-Stage Rehabilitation? A Systematic Review

**DOI:** 10.3390/ijerph192315893

**Published:** 2022-11-29

**Authors:** Maciej Kochman, Marta Kasprzak, Aleksandra Kielar

**Affiliations:** Physiotherapy Department, Institute of Health Sciences, College of Medical Sciences, University of Rzeszów, Marszałkowska 24, 35-215 Rzeszów, Poland

**Keywords:** physiotherapy, rehabilitation, physical activity, ACL reconstruction, early phase

## Abstract

Despite the restoration of the mechanical stability of the knee joint after ACL reconstruction (ACLR), patients often experience postoperative limitations. To our knowledge, there are no systematic reviews analyzing additional physiotherapy interventions implementing standard rehabilitation programs in the early postoperative phase after ACLR. The objective of this study was to analyze the additional physiotherapy interventions implemented in standard rehabilitation programs that improve early-stage ACLR rehabilitation. For this systematic review, we followed the PRISMA guidelines. In March 2022 we conducted a literature review using electronic databases. Primary outcomes were pain, edema, muscle strength, ROM, and knee function. The risk of bias and scientific quality of included studies were assessed with the RoB 2, ROBINS-I and PEDro scale. For the review, we included 10 studies that met the inclusion criteria (total *n* = 3271). The included studies evaluated the effectiveness of Kinesio Taping, Whole-body vibration, Local Vibration Training, Trigger Point Dry Needling, High Tone Power Therapy, alternating magnetic field, and App-Based Active Muscle Training Program. Most of the additional physiotherapy interventions improved pain, edema, ROM, knee muscle strength, or knee function in early-stage postoperative ACL rehabilitation. Except for one study, no adverse events occurred in the included studies, which demonstrates the safety of the discussed physiotherapy interventions. Further in-depth research is needed in this area.

## 1. Introduction

The anterior cruciate ligament (ACL) plays an important role in the kinematics of the knee joint, limiting the anterior translation of the tibia and stabilizing the knee joint [1]. It also contains mechanoreceptors whose task is to maintain neuromuscular control of the joint [2]. At the same time, it is a structure whose damage is one of the most common and serious locomotor injuries [3]. The consequence of ACL rupture is a disturbance in the biomechanics of the joint, leading to the development of abnormal movement patterns and chronic instability that leads to the loss of function during dynamic tasks and may cause secondary damage to the menisci and cartilage [3,4]. A complete ACL rupture is usually an indication for reconstruction. It should be emphasized that the prognosis of patients after surgery is strictly dependent on the implemented rehabilitation [5]. Despite the restoration of mechanical stability of the joint after ACL reconstruction (ACLR), patients often experience pain [6], swelling [7], reduced muscle strength [8], or reduced range of motion for a long time after surgery, which can cause functional limitations and lead to a deterioration of their quality of life [9]. Standard physiotherapy is often insufficient for patients after ACL reconstruction, therefore, there is a need to improve existing rehabilitation programs [10], especially in the early postoperative phase. In the scientific literature, there are many systematic reviews assessing the effects of physiotherapy after ACL reconstruction; however, to the best of our knowledge, there are no systematic reviews analyzing additional physiotherapy interventions implementing standard rehabilitation programs in the early postoperative phase. Previous systematic reviews included studies evaluating the effects of physiotherapy in both early and late-stage ACL rehabilitation [11,12,13,14,15], preoperative rehabilitation [16,17], or both preoperative and postoperative rehabilitation [18]. In this study, we focused only on the early phase of ACL rehabilitation as it may be limited by postoperative complications such as pain or edema and these are often a challenge for both clinicians and patients in the first postoperative weeks [19] as swelling causes a decrease in the quadriceps strength through arthrogenic muscle inhibition [20] and the pain complicates exercising and functional performance [20,21]. By conducting this review, we wanted to answer the following question: What physiotherapy interventions improve pain, edema, knee range of motion, muscle strength, and function after ACL reconstruction in the early phase? Therefore, the objective of this study was to analyze the additional physiotherapy interventions implemented in standard rehabilitation programs that improve early-stage ACL reconstruction rehabilitation.

## 2. Materials and Methods

### 2.1. Protocol Design

For this systematic review, we followed the Preferred Reporting Items for Systematic Reviews and Meta-Analyses (PRISMA) guidelines [22,23]. This study was registered with the PROSPERO database on 25 April 2022 with ID number: CRD42022320710.

### 2.2. Search Strategy

In March 2022 we conducted a literature review using PubMed, Medline, Physiotherapy Evidence Database (PEDro), Science Direct, Scopus, and Web of Science (WOS). Keyword sets used in the database search are presented in Table 1. Detailed search equations are included in Appendix A.

The search terms within each group were combined with the ‘OR’ operator; the ‘AND’ operator was used to combine the results from all four groups to obtain the final yield. A systematic literature search was performed by two authors (M.Ka. & A.K.) independently and included clinical trials published from 2012 to 2022.

### 2.3. Study Selection and Eligibility Criteria

After the selection of keywords, all document sets were analyzed and duplicates between different databases were removed. Two review authors (M.Ka. and A.K.) selected potentially eligible articles by reviewing the title and abstract of each document. In the case of disagreement, a consensus was sought between the above authors. If consensus was not reached, the report was included for full-text evaluation. After obtaining the full articles, both authors (M.Ka. and A.K.) independently performed study selection using inclusion and exclusion criteria. In the case of disagreement, a consensus was sought between the two above authors; further disagreement was arbitrated by a corresponding author (M.Ko.). For this systematic review, we included only RCTs or CTs, full-text studies written in English and containing information on the use of additional physiotherapy interventions on knee pain, swelling, strength, ROM, and function in the early phase after ACL reconstruction. We excluded studies that were not RCTs and CTs and did not concern the early phase of rehabilitation after ACL reconstruction. We also excluded studies in which there was no control group, if the control group consisted of non-ACL-reconstructed persons, if more than two groups participated in the study, and if physiotherapy intervention was introduced before the reconstruction. Studies that reported only qualitative data or unanalyzed quantitative data were excluded. Where necessary, the authors were contacted for additional information to obtain incomplete information. If the authors could not be contacted, the article was excluded from the review.

### 2.4. Data Extraction and Synthesis

Two review authors (M.Ka. and A.K.) extracted and prepared data for synthesis from each study using the same form following the PICO model: Participants (characteristics of the study population including the number of participants, gender and age, ACL reconstruction method and time after reconstruction), Intervention (type and duration of the intervention), Comparison between groups and Outcome measures (based on knee pain, swelling, strength, ROM and function). We observed a significant lack of homogeneity between study designs, type and duration of interventions and finally the type and timing of outcome measures. It was not possible to pool the data into a statistical meta-analysis also due to the imprecision of the estimates; therefore, we conducted a narrative synthesis with a summary of the findings (a synthesis without meta-analysis; SWiM) [24]. We presented the strength of evidence of outcomes with the statistically significant results (*p* < 0.05) and calculated Cohen’s d effect sizes for statistically significant outcomes, where means and standard deviations were available and marked them as trivial (effect size greater than 0 and lower than 0.2), small (effect size greater than or equal to 0.2), medium (effect size greater than or equal to 0.5) or large (effect size and greater than or equal to 0.8) [25,26]. We also assessed the certainty of the evidence according to the Grading of Recommendations, Assessment, Development and Evaluations (GRADE) using the GRADEpro GDT tool considering the following domains: risk of bias, inconsistency, indirectness, imprecision and overall certainty level. The overall evidence was rated as very low, low, moderate, or high [24]. Data were extracted, synthesized and tabulated into standardized tables developed for this review. The studies were grouped by intervention.

### 2.5. Evaluation of Study Quality and Risk of Bias

The study quality and risk of bias assessment were performed by two review authors (M.Ka. and A.K.) using the PEDro scale and the Cochrane Risk of Bias Tools for Randomized (RoB 2) and Nonrandomized Clinical Studies (ROBINS-I). In the case of any disagreement, the final decision was made by the third author (M.Ko.). The PEDro scale is used to assess the methodological quality of clinical trials included in systematic reviews across physiotherapy, health and medical research. The PEDro Scale assesses 11 items related to the study’s internal validity and statistical reporting, except for the first one (eligibility criteria), which is not computed in the total score. Each item is scored as either present (1) or absent (0), leading to a maximum score of up to 10. Higher scores indicate superior methodological quality. According to Cashin, studies can be divided into four quality groups based on the achieved PEDro score and level of methodological quality: <4 are considered ‘poor’, 4–5 are considered ‘fair’, 6–8—are considered ‘good’, 9–10—are considered ‘excellent’ [27]. The RoB 2 tool assesses bias in five distinct domains: bias arising from the randomization process, bias due to deviations from intended interventions, bias due to missing outcome data, bias in the measurement of the outcome, and bias in the selection of the reported result. The judgments within each domain lead to an overall risk of bias of: “low risk of bias,” “some concerns,” or “high risk of bias” [28]. The ROBINS-I tool views each study as an attempt to emulate a hypothetical pragmatic randomized trial and assesses seven domains through which bias might be introduced: bias due to confounding, bias in the selection of participants into the study, bias in classification of interventions, bias due to deviations from intended interventions, bias due to missing data, bias in the measurement of outcomes, and bias in the selection of the reported result. The judgments within each domain carry forward to an overall risk of bias of: “Low risk of bias”, “Moderate risk of bias”, “Serious risk of bias”, “Critical risk of bias” or “No information” [29].

## 3. Results

### 3.1. Selection of Included Studies

**Figure 1:**
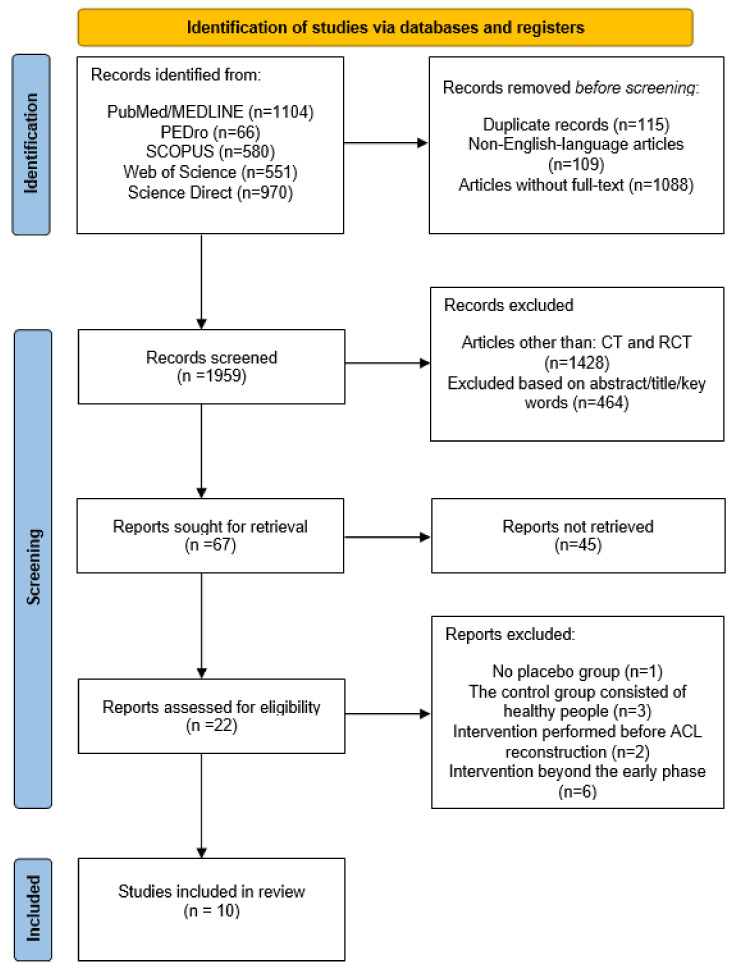
Flow chart with summary of database search.

The initial search identified 3271 studies; after screening and eligibility assessment 10 studies (nine RCTs and one CT) that met the inclusion criteria were included. The PRISMA flow chart for this systematic review is presented in Figure 1.

### 3.2. Characteristics of the Included Studies

Included studies assessed the effectiveness of Kinesio Taping (KT; three studies) [30,31,32], Whole body vibration (WHB; two studies) [33,34], Local Vibration Training (LVT; one study) [35], trigger point dry needling (TrPDN; one study) [36], High Tone Power Therapy (HiToP; one study) [37], alternating magnetic field (one study) [38], and app-based active muscle training program (App-Based Serious Gaming; one study) [39]. The included studies were conducted in Italy [32,34], France [31,35], Poland [37,38], Germany [33,39], Spain [36] and Turkey [30]. In this review, we included two articles published in 2021 by the same author. In order to avoid confusion, we have marked them as Ogrodzka-Ciechanowicz et al. I (2021) and Ogrodzka-Ciechanowicz et al. II (2021).

### 3.3. Data Synthesis and Certainty of Evidence

In the included studies, we found a high level of heterogeneity regarding different interventions, outcomes, and measures but also imprecision of estimates, which did not allow us to undertake a detailed meta-analysis. Knee function was the most measured outcome in eight studies [30,32,33,34,35,36,37,39]; pain [30,31,32,36,37,39], ROM [30,32,33,36,37,38], and strength of extensor muscles [30,33,34,35,37,39] were evaluated in six studies; edema in five studies [30,32,37,38,39] and strength of flexor muscles in three studies [30,33,34]. A subgroup analysis with a synthesis of the outcomes is presented in Table 2.

We assessed the overall level of certainty of the evidence for all the evaluated outcomes as ‘very low’. The outcomes were assessed with ‘serious concerns’ in every domain except for Risk of Bias (we had ‘no serious concerns’ for pain and edema evaluation). The other outcomes were downgraded mainly because of a lack of allocation concealments, intention-to-treat analysis, or blinding patients, healthcare providers and assessors. We also had ‘serious concerns’ about inconsistency due to statistical heterogeneity. We also had ‘serious concerns’ in indirectness across all evaluated outcomes. The outcomes were downgraded mainly because of different types of interventions but also population differences (graft type, different enrollment time). We also had ‘serious concerns’ for imprecision across all evaluated outcomes as they were downgraded mainly because of small sample sizes. The certainty of evidence assessment is presented in Table 3.

### 3.4. Quality and Risk of Bias Assessment of the Included Studies

The studies included in the analysis ranged from 4 to 9 on the PEDro scale. According to the PEDro score, four studies [31,32,33,35] were considered ‘fair’, five studies [30,34,36,37,39] were ‘good’ and one study [38] was ‘excellent’; 9/10 articles were randomized (90%), but only three had a concealed allocation conducted (30%). All reported publications scored particularly poorly in the blinding of subjects, therapists and assessors, potentially resulting in a higher risk of bias. The methodological quality results of the included studies are presented in Table 4. According to the RoB 2 analysis, 22.2% of included studies [36,38] presented an overall ‘low risk of bias’ and 11.1% of studies [35] presented ‘high risk of bias’. We had ‘some concerns’ in overall bias in 66.7% of studies [30,32,33,34,37,39]. When analyzing the domains of each study individually, 100% of studies presented a ‘low risk of bias’ in the randomization process. We had ‘some concerns’ of a bias in 66.7% of the studies on the deviations from intended interventions, while ‘low risk of bias’ we observed in 33.3% of the studies. In the missing outcome data domain, we observed a ‘low risk of bias’ in 88.9% of the studies and a ‘high risk of bias’ in 11.1% of the studies. We had ‘some concerns’ of a bias in 66.7% of studies of the measurement of the outcome, while ‘low risk of bias’ we observed in 33.3% of the studies; 100% of the studies presented a ‘low risk of bias’ in the selection of the reported result. These results are shown in Table 5. According to the ROBINS-I analysis, we observed a ‘moderate risk of overall bias’ in the study of Laborie et al. These results are shown in Table 6.

### 3.5. Participants

A total of 379 patients (including 74 females and 271 males; Pistone et al. did not reveal details of the participants’ gender) took part in the 10 included studies; 189 participants (pooled mean age 28.7 ± 7.40) were allocated to the experimental group and 190 were controls (pooled mean age 29.1 ± 7.9). Study participants underwent ACL reconstruction with hamstring tendon graft [31,34,37,38,39], patellar graft [33] or other types of the graft [30,32,35,36] and were enrolled in the study immediately postoperatively [31,39], within the first postoperative week [30,32,35,38], within a second postoperative week [33,37] or between second and forth postoperative week [34,36].

### 3.6. Intervention

Included studies varied significantly regarding the intervention type and its duration. As mentioned above, three included studies assessed the effectiveness of KT [30,31,32], two studies assessed WHB [33,34], one study assessed LVT [35], one study assessed TrPDN [36], one study assessed HiToP [37], one study assessed alternating magnetic field [38], and one study assessed app-based active muscle training program (App-Based Serious Gaming) [39]. There were one-time [31,36] short-term [30,37] and long-term interventions [31,33,34,35,37,39]. In 6/10 of included studies, there was no follow-up [31,32,33,35,37,38], in the other studies, the follow-up ranged from 3 weeks to 10 weeks [30,34,36,39].

### 3.7. Comparison

Participants in all included studies were divided into two groups: experimental and control. Control groups received standard rehabilitation programs or were treated according to the physician’s instructions [38], postoperative treatment protocol [39], or the department’s usual anesthesia-analgesia protocol [31]. The same standard procedures were received by the experimental groups in all studies, except for one study [33], where the experimental group received the WBV protocol of the same duration as the standard rehabilitation program in the control group. Measurements were taken at the same time and with the same instruments for both experimental and control groups.

### 3.8. Outcomes

As mentioned above, in this review we focused on pain, edema, knee ROM, function, and muscle strength evaluation. In the included studies the measurements were taken daily [30,31,38], weekly [32,33,35,36,39], or monthly [34,37]. Outcomes were measured with the same instruments in experimental and control groups within individual studies but differed between included studies: VAS (pain), tailor’s tape (edema), ROM (goniometer), dynamometer or force transducer (muscle strength), and Lysholm test, Tegner scale, WOMAC or KOOS (knee function).

### 3.9. Summary of Reviewed Studies

A summary of the reviewed studies is presented in Table 7, Table 8 and Table 9 including the authors and year of publication, design of the study, method of randomization, and characteristics of the study population (number of participants, gender and age). The tables also include the ACLR method, enrollment time since the surgery, type and duration of the intervention, outcome measures, and finally main outcomes.

### 3.10. Kinesio Taping

We included three studies that evaluated the effectiveness of KT during the early postoperative phase of rehabilitation after ACL reconstruction. Balki et al. (2016) investigated the effect of KT on swelling, pain, muscle strength, ROM, and subjective functions. For this study, 30 men aged 18 to 39 years were recruited on the fourth postoperative day after ACL reconstruction with hamstring tendon autograft and tibialis posterior or peroneus longus allograft. The population was randomly assigned to two groups: an experimental (15 men) with two muscle and lymphatic KT techniques every 5 days during a 10-day period that implemented a 12-week standard rehabilitation program and a control group (15 men) with two placebo KT techniques every 5 days for 10 days that implemented a 12-week rehabilitation program. Measurements were taken on the fourth day postoperatively, after 5 and 10 days post-KT application, and as a follow-up after the first and third month postoperatively. Compared to the control group, the experimental group showed significant improvements in knee swelling, pain measurements, hamstring muscle strength, and knee flexion ROM (*p* < 0.05) but not subjective functions, knee extension, and extensor muscle strength (*p* > 0.05).

Laborie et al. (2015) evaluated the efficacy of KT in early postoperative pain after ACL reconstruction. 57 patients (44 men and 13 women) after ACL reconstruction with hamstring tendon graft were recruited for the study immediately after surgery. The population was divided into two groups: the experimental (21 men and 7 women) with muscle/lymphatic KT technique for 3 days implemented in the postoperative anesthesia-analgesia protocol and the control group (21 men and 7 women) with the anesthesia-analgesia protocol only. Measurements were taken at baseline and on the first, second and third day after KT application. In this study, Kinesio Taping did not show efficacy in early postoperative pain compared to the control group (*p* > 0.05).

Labianca et al. (2021) evaluated the effect of KT on reducing postoperative knee edema and pain, improving ROM and muscle mass recovery. For this study, 52 men after ACL reconstruction with gracilis and semitendinosus tendon autograft aged 18–45 were recruited on the second postoperative day. The study population was randomly assigned to the experimental group (26 men) with muscle and lymphatic KT application every 5 days for 4 weeks that implemented the standard 4-week rehabilitation program and the control group (26 men) with the standard 4-week rehabilitation program only. Measurements were taken at the end of the second and fourth postoperative weeks. The KT group showed improvement in pain intensity and edema reduction (*p* < 0.05) but not in ROM recovery and knee function (*p* > 0.05).

### 3.11. Vibration Training

We included two studies that evaluated the effectiveness of WBV and one study that evaluated the effectiveness of LVT during early postoperative phase rehabilitation after ACL reconstruction. Berschin et al. (2014) evaluated the effect of WBV training on neuromuscular performance (strength and coordination) in the short term after ACLR. This study included 40 patients (29 men and 11 women) after ACL reconstruction with patellar tendon graft aged 25–39 on the 14th day after the operation. The study population was randomly divided into two groups: a WBV exercise group (experimental group, 14 men and 6 women) with an 8-week WBV protocol training and a control group (15 men and 5 women) with 8 weeks of a standard rehabilitation program. Measurements were taken at baseline, fifth, eighth and eleventh week postoperatively. Compared to the control group, the WBV group did not show improvement in ROM, muscle strength, or knee function (*p* > 0.05).

Pistone et al. (2016) investigated the effect of early WBV training at an optimal frequency on maximal strength and balance after ACLR in the short term. For this study, 34 patients (the author did not reveal the gender or age range of the participants) after ACL reconstruction with a hamstring tendon autograft were recruited 1 month after surgery. The study population was randomly assigned to two groups: an experimental group (17 patients) with 4 weeks of WBV training implemented in the standard 4-week rehabilitation program and a control group (17 patients) with the standard 4-week rehabilitation program. Measurements were taken at baseline, and the second and third months postoperatively. Compared to the control group, the WBV group showed improvement in knee flexor strength symmetry and knee function (*p* < 0.05) but not in knee extensor strength symmetry (*p* > 0.05).

Coulondre et al. (2021) evaluated the effect of LVT on quadriceps strength in the early post-ACL reconstruction period. Twenty-three patients (13 men and 10 women) after ACL reconstruction with hamstring or patellar tendon autograft aged 18–50 were recruited into the study within one postoperative week (enrollment time was established individually). The study population was randomly assigned to two groups: experimental (6 men and 5 women) with 24 LVT sessions implemented in 24 standard rehabilitation sessions (approximately 10 weeks) and control (7 men and 5 women) with 24 sessions of the standard rehabilitation program (approximately 10 weeks) only. Measurements were taken individually for each participant: first at baseline and second after the last session. Compared to the control group, the LVT group showed improvement in PRE-POST extensor muscle strength changes (*p* < 0.05) but not in limb strength symmetry and functional tests (*p* > 0.05).

### 3.12. Miscellaneous Interventions

Four studies concerned a variety of other physiotherapy interventions. Velázquez-Saornil et al. evaluated the effect of TrPDN on pain intensity, ROM, stability, and functionality after ACL reconstruction. For this study, 44 patients (28 men and 16 women) after ACL reconstruction with patellar or hamstring tendon grafts aged 19–51 were recruited within 12–19 postoperative days. The study population was randomly assigned to the experimental group (16 males and 6 females) with a one-time vastus medialis trigger point dry needling intervention implemented in a 5-week rehabilitation program and the control group (12 males and 10 females) with a 5-week rehabilitation program only. Measurements were taken at baseline, immediately after the intervention, after 1 day, 1 week and 5 weeks after the intervention. Unfortunately, the TrPDN group showed an increase in pain (only immediately after the intervention; *p* < 0.05) but also in ROM and knee function (*p* < 0.05).

In the first study, Ogrodzka-Ciechanowicz et al. evaluated the effectiveness of quadricep muscle electrostimulation with HiToP therapy on pain and functional level in patients after ACL reconstruction. Thirty-five male patients after ACL reconstruction with hamstring tendon graft aged 21–50 were recruited on the eleventh postoperative day for the study. The study population was randomly divided and assigned to one of the following groups: experimental (17 men) with 1 h of HiToP therapy on the quadriceps muscle implemented in each rehabilitation program session for 6 months and control (18 men) with 6 months of the rehabilitation program. Measurements were taken 2 days before the first intervention and 6 months after surgery. The HiToP group showed an improvement in extensor muscle strength, knee and thigh circumferences, knee extension, and knee function (*p* < 0.05) but not pain (*p* > 0.05).

The second study by Ogrodzka-Ciechanowicz et al. evaluated the efficiency of the alternating magnetic field in the resorption of postoperative joint effusion in patients after ACL reconstruction. For this study, 38 patients (28 men and 10 women) were recruited after ACL reconstruction with semitendinosus tendon autograft aged 18–40 on the first postoperative day. The study population was randomly assigned into two groups: the experimental (15 men and four women) with 10 days of 30-minute alternating magnetic field therapy each day implemented according to the physician’s postoperative instructions and the control group (13 men and 6 women) with 10 days of 30-minute placebo magnetic field therapy each day implemented according to physician’s postoperative instructions. Measurements were taken at baseline and daily after therapy for 11 days. Compared to the control group, the alternating magnetic field group did not show improvement in knee joint effusion and AROM (*p* > 0.05).

Clausen et al. investigated the effect of app-based active muscle training (app-based serious gaming) on strength in the initial postoperative phase after ACLR. For this pilot study, 26 patients (12 men and 14 women) were immediately recruited after ACL reconstruction with a hamstring graft. The study population was randomly divided into an experimental group (6 men and 8 women) with 3 weeks of serious app-based games (5 times a day) implemented in the rehabilitation program and a control group (6 men and 6 women) with 3 weeks of rehabilitation program only. Measurements were taken before and 6 weeks after the operation. App-based active muscle training showed improvement in maximum strength (*p* < 0.05) but not in knee function and pain (*p* > 0.05).

## 4. Discussion

This systematic review aimed to evaluate the additional physiotherapy interventions implemented in standard rehabilitation programs that could improve ACL reconstruction rehabilitation in the early postoperative phase in terms of pain, swelling, function, muscle strength, and range of motion improvement. We included ten studies in this review, three of them assessed the effectiveness of KT, three evaluated vibration training, and four miscellaneous interventions. We encountered many problems in the attempt to evaluate the articles. The methodological quality of the included studies varied and ranged from 4 to 9 on the PEDro scale; we also had concerns according to the risk of bias assessment. Unfortunately, the protocols of studies included in this review showed a high level of heterogeneity regarding study designs, study population (graft type), enrollment time, intervention type and duration, as well as methods and timing of patients’ outcome measures. Therefore, the results of these investigations were difficult to compare. We need to draw attention to the lack of standardized methodology, validated assessment methods and outcome measures. Reports evaluating the effectiveness of KT are contradictory and various reasons may have played a role in this intervention such as different duration times and techniques of KT application used in the study but also graft type used for ACL reconstruction. In a study by Laborie et al., the duration of KT application was the shortest (3 days), which could be the main factor that resulted in no significant pain improvement in the KT group. The remaining studies confirmed a significant improvement in both pain and swelling after the use of KT (*p* < 0.05). The studies also assessed knee function, range of motion and muscle strength, but no significant difference was found between the study groups (*p* > 0.05). This has also been shown in other studies [40,41]. Only the study by Balki et al. showed a significant improvement in the flexion ROM and the strength of the knee flexor muscles (*p* < 0.05). Reports evaluating WBV training are also contradictory as Berschin et al. did not prove the effectiveness of this method in the range of motion, knee function and muscle strength (*p* > 0.05), while Pistone et al. demonstrated a significant improvement in the function and strength of the knee flexor muscles (*p* < 0.05). This could be explained by the fact that in the second study, participants were enrolled in the study later (one month after the ACLR), which may indicate that WBV is more effective in the later phase of rehabilitation after ACLR; this has also been shown in other studies [40,41,42,43]. Other reasons explaining this discrepancy could be differences in the protocol (WBV vs. WBV plus standard rehabilitation program) or graft type (patellar tendon vs. hamstring graft). Coulondre et al. showed that LBV improved knee extension muscle strength (*p* < 0.05) but did not improve knee function (*p* > 0.05). Similarly, Ming et al. reported that LVT improved quadriceps muscle strength and knee ROM [44]. Other studies should be evaluated with caution, as they are individual studies investigating individual interventions, so they should not lead to far-reaching conclusions. We recommend treating this analysis as a starting point for further research in this field. Velázquez-Saornil et al. confirmed the effectiveness of TrPDN in improving knee range of motion and function, as well as worsening pain (on one measurement only, immediately after the intervention). Additionally, due to an adverse event that occurred in the experimental group, one subject was excluded from the study. Ortega-Cebrian et al. evaluated the effect of dry needling during late-stage rehabilitation of patients reconstructed with ACL and confirmed that this intervention improved knee ROM flexion and pain in the later phase of ALCR rehabilitation [45]. In the first study, Ogrodzka-Ciechanowicz et al. confirmed the effectiveness of HiToP in improving the swelling, range of motion, knee function, and strength of the knee extensor muscles (*p* < 0.05), but not pain (*p* > 0.05). In the second study, Ogrodzka-Ciechanowicz et al. confirmed the ineffectiveness of alternating magnetic fields in edema and knee range of motion (*p* > 0.05). The last study evaluated the effectiveness of an app-based active muscle training program in improving pain, swelling, function, and strength in knee extensor muscles. Clausen et al. confirmed the effectiveness of this intervention on the strength of the extensor muscle of the knee joint (*p* < 0.05).

### 4.1. Practical Implications

This systematic review summarizes the latest research (from 2014 onwards) on the efficacy of physiotherapy interventions supplementing standard rehabilitation programs or postoperative treatment protocols in the early-stage ACL reconstruction phase. Therefore, it may be considered a recommendation for clinical physiotherapists to improve existing rehabilitation protocols and inspiration for other scientists to deepen research in this area.

### 4.2. Study Limitations

We must admit that this systematic review has some limitations. Firstly, the level of methodological quality of the analyzed studies was rather poor with regard to mainly blinding of the participants, investigators and assessors; therefore, there was potential for selection and interpretation bias. Secondly, the high level of clinical heterogeneity in the studies caused limitations with data analysis (different types of graft, enrollment time, type and duration of intervention and finally the timing of outcome measures); therefore, a meta-analysis could not be performed. Finally, despite the performance of a study search and selection independently by two authors, there is a possibility that some related studies might not have been included in this review.

## 5. Conclusions

Most of the additional physiotherapy interventions discussed in this review improved pain, edema, ROM, knee muscle strength, or knee function in the early-stage postoperative ACL rehabilitation. Except for one study [36], no adverse events occurred in the included studies, which demonstrates the safety of the discussed physiotherapy interventions. It should also be emphasized that in some studies no significant differences were found between the experimental and the control groups; however, intra-group comparisons in both groups showed an improvement from the baseline. As some reports are contradictory, we recommend treating them with caution and not drawing solid conclusions. Further in-depth research is needed to evaluate the physiotherapy interventions in this review, taking into account standardized methodology, a larger study population, a longer duration of the study including the follow-up, validated assessment methods, and outcome measures.

## Figures and Tables

**Table 1:** Keyword sets used in the database search.

Anterior Cruciate Ligament Reconstruction	Rehabilitation	Phase	Measure
ACL Reconstruction ACLR Repair Surgery	Physiotherapy Physical therapy Training Exercise Intervention Treatment Standard rehabilitation program	Acute phase Early phase Initial postoperative phase	Pain Effusion Edema Swelling Range of motion ROM AROM Muscle strength Knee function Knee activity

**Table 2:** Subgroup analysis with synthesis of the outcomes.

Study	Intervention	Outcome					
		Pain	Edema	ROM	Knee Function	Strength of Flexors	Strength of Extensors
Balki et al. (2016) [30]; RCT	KT	+	+	+ (l)	-	+ (l)	-
Laborie et al. (2015) [31]; nRCT	KT	-					
Labianca et al. (2022) [32]; RCT	KT	+ (s)	+ (l, t)	-	-		
Berschin et al. (2014) [33]; RCT	WBV			-	-	-	-
Pistone et al. (2016) [34]; RCT	WBV				+ (l)	+	-
Coulondre et al. (2021) [35]; RCT	LVT				-		+ (l)
Velázquez-Saornil et al. (2017) [36]; RCT	TrPDN	* (l)		+ (s, l)	+ (m, l)		
Ogrodzka-Ciechanowicz et al. I (2021) [37]; RCT	HiToP	-	+ (l)	+ (l)	+ (l)		+ (l)
Ogrodzka-Ciechanowicz et al. II (2021) [38]; RCT	Alternating magnetic field		-	-			
Clausen et al. (2020) [39]; RCT	App-based serious gaming	-	-		-		+ (m)

+, significant difference in favor of experimental versus control group; *, significant difference in favor of control versus experimental group; -, no difference or no improvement between groups; (t), (s), (m), (l), Cohen’s d effect size: trivial, small, medium or large (only where means and standard deviations were available).

**Table 3:** Certainty of evidence assessment.

No. of Studies	Study Design	Risk of Bias	Inconsistency	Indirectness	Impercision	Certainty
Pain (assessed with: VAS)						
5	randomised trials	Not Serious	Serious	Serious	Serious	⨁◯◯◯ Very low
Edema (assessed with: tailor’s tape)						
5	randomised trials	Not Serious	Serious	Serious	Serious	⨁◯◯◯ Very low
Range of motion—ROM (assessed with: goniometer)						
6	randomised trials	Serious	Serious	Serious	Serious	⨁◯◯◯ Very low
Knee function (assessed with: Lysholm test/WOMAC/Tegner scale/KOOS)						
8	randomised trials	Serious	Serious	Serious	Serious	⨁◯◯◯ Very low
Strength of flexors (assessed with: Dynamometer/force transducer)						
3	randomised trials	Serious	Not Serious	Serious	Serious	⨁◯◯◯ Very low
Strength of extensors (assessed with: Dynamometer/force transducer)						
6	randomised trials	Serious	Serious	Serious	Serious	⨁◯◯◯ Very low
Pain (assessed with: VAS)						
1	nRCT	Serious	Serious	Serious	Serious	⨁◯◯◯ Very low

**Table 4:** PEDro score quality assessment.

	Balki et al. (2016) [30]	Laborie et al. (2015) [31]	Labianca et al. (2022) [32]	Berschin et al. (2014) [33]	Pistone et al. (2016) [34]	Coulondre et al. (2021) [35]	Velázquez-Saornil et al. (2017) [36]	Ogrodzka-Ciechanowicz et al. I (2021) [37]	Ogrodzka-Ciechanowicz et al. II (2021) [38]	Clausen et al. (2020) [39]
Eligibility Criteria *	No	Yes	No	Yes	Yes	Yes	No	Yes	Yes	Yes
Randomly allocated	Yes	No	Yes	Yes	Yes	Yes	Yes	Yes	Yes	Yes
Concealed Allocation	No	No	No	No	No	No	Yes	Yes	Yes	No
Similar groups at baseline	Yes	Yes	No	Yes	Yes	Yes	Yes	Yes	Yes	Yes
Blinding of Subjects	Yes	No	No	No	No	No	No	No	Yes	No
Blinding of Therapists	No	No	No	No	No	No	No	Yes	Yes	No
Blinding of Assessors	Yes	No	No	No	No	No	Yes	No	No	No
Data from >85% of Subjects	Yes	Yes	Yes	Yes	Yes	No	Yes	Yes	Yes	Yes
Intention-to-Treat Analysis	No	Yes	No	No	Yes	No	Yes	No	Yes	Yes
Statistical comparision	Yes	Yes	Yes	Yes	Yes	Yes	Yes	Yes	Yes	Yes
Measures of Variability	Yes	Yes	Yes	Yes	Yes	Yes	Yes	Yes	Yes	Yes
Final score	7/10	5/10	4/10	5/10	6/10	4/10	8/10	7/10	9/10	6/10

* Criterion number 1 is not used to calculate the PEDro score [27].

**Table 5:** Risk of Bias assessment of RTCs.

Risk of Bias for Included Randomized Trials (RoB 2)						
Study	Randomization Process	Deviations from the Intended Interventions	Missing Outcome Data	Measurement of the Outcome	Selection of the Reported Result	Overall
Balki et al. (2016) [30]	Low risk	Some concerns	Low risk	Low risk	Low risk	Some concerns
Labianca et al. (2022) [32]	Low risk	Some concerns	Low risk	Some concerns	Low risk	Some concerns
Berschin et al. (2014) [33]	Low risk	Some concerns	Low risk	Some concerns	Low risk	Some concerns
Pistone et al. (2016) [34]	Low risk	Low risk	Low risk	Some concerns	Low risk	Some concerns
Coulondre et al. (2022) [35]	Low risk	Some concerns	High risk	Some concerns	Low risk	High risk
Velázquez-Saornil et al. (2017) [36]	Low risk	Low risk	Low risk	Low risk	Low risk	Low risk
Ogrodzka-Ciechanowicz et al. I (2021) [37]	Low risk	Some concerns	Low risk	Some concerns	Low risk	Some concerns
Ogrodzka-Ciechanowicz et al. II (2021) [38]	Low risk	Low risk	Low risk	Low risk	Low risk	Low risk
Clausen et al. (2020) [39]	Low risk	Some concerns	Low risk	Some concerns	Low risk	Some concerns

**Table 6:** Risk of Bias assessment of nRTC.

Risk of Bias Included Nonrandomized Trials (ROBINS-I)								
Study	Confounding	Selection of Participants	Classification of Interventions	Deviations from Intended Interventions	Missing Data	Measurement of Outcomes	Selective Reporting	Overall
Laborie et al. (2015) [31]	Low	Low	Moderate	Low	Moderate	Moderate	Low	Moderate

**Table 7:** Summary of reviewed studies on KT.

Author	Balki et al. (2016) [30] RCT	Laborie et al. (2015) [31] CT	Labianca et al. 2022 [32] RCT
Method of randomization	Randomization table	No randomization	Online randomizer tool
Study population	N = 30; Experimental group N = 15 (15M; aged 22–37; mean age 28.60 ± 4.50), Control group N = 15 (15M; aged 18–39; mean age 27.66 ± 7.45).	N = 57 (44M, 13F); Experimental group N = 28 (21M, 7F; mean age 29.2 ± 8.6); Control group N = 29 (23M, 6F; mean age 32.6 ± 9.1)	N = 52 (M; aged 18–45); Experimental group N = 26 (26M; mean age 28.5 ± 5.3); Control group N = 26 (26M; mean age 29.2 ± 4.6)
ACLR method	Hamstring tendon autograft and tibialis posterior or peroneus longus allograft.	Hamstring tendon graft	Gracilis and semitendinosus tendon autograft
Enrollment time	4th day postoperatively	Immediately postoperatively	2nd day postoperatively
Intervention	2 muscle and lymphatic KT techniques every 5 days during a 10-day period plus 6 week rehabilitation program vs. 2 placebo KT techniques every 5 days during a 10-day period plus 6 week rehabilitation program	3 days of muscle/lymphatic KT technique plus anesthesia-analgesia protocol vs. 3 days of anesthesia-analgesia protocol	Muscle and lymphatic KT application every 5 days for 4 weeks plus standard 4 week rehabilitation program vs. standard 4 week rehabilitation program
Outcome measures	On 4th day (baseline; before KT application) and after 5 and 10 days after KT application: Pain (VAS); swelling (circumferences; tailor’s tape); ROM of knee flexion and extension (goniometer); hamstring and quadriceps strength (dynamometer). Follow up after 1 and 3 months: subjective functions (modified Cincinnati (30-point), Lysholm and Tegner tests).	At baseline (in the evening and at night) and on days: 1, 2, 3 after KT application: knee pain intensity (VAS); analgesia intake, awakening due to pain, postoperative discomfort, allergic reaction to KT, overall patient satisfaction (online self-assessment survey).	At the end of 2nd and 4th postoperative weeks: pain (VAS scale), edema (girth; measured at the mid patella), muscle mass (thigh circumference; measured 10 cm above the upper edge of the patella), passive knee ROM and knee function (Tegner-Lysholm Knee Scale and Knee Injury and Osteoarthritis Outcome Score (KOOS))
Main outcomes	Significant improvements in experimental vs. control group in knee swelling, pain and hamstring muscle strength (6.33 ± 1.54 vs. 5.13 ± 1.40) on the 5th day (*p* < 0.05); in knee flexion ROM (76.80 ± 14.85 vs. 60.13 ± 8.79), night pain, knee swelling and hamstring muscle strength (9.86 ± 2.32 vs. 7.53 ± 2.16) on the 10th day (*p* < 0.05) No significant improvement in experimental vs. control group in knee function, extension and extensor muscle strength (*p* > 0.05).	No significant difference in experimental vs. control group for knee pain intensity, evolution of pain and overall satisfaction (*p* > 0.05).	Significant improvement in experimental vs. control group in pain intensity at week 2 (3.2 ± 1.6 vs. 4.7 ± 1.9; *p* = 0.029) and edema reduction at week 2 (−6.0 ± 2.2 vs. −0.75 ± 0.5; *p* = 0.007) and week 4 (−7.6 ± 2.9 vs. −2.75 ± 1.4; *p* = 0.006). No significant improvement in experimental vs. control group in thigh circumference, ROM recovery, and knee function (*p* > 0.05)

**Table 8:** Summary of reviewed studies on WBV and LVT.

Author	Berschin et al. (2014) [33] RCT	Pistone et al. 2016 [34] RCT	Coulondre et al. 2021 [35] RCT
Method of randomization	Computer generated numbers	Block randomization program	REDCap Web application
Study population	N = 40 (29M and 11F). Experimental group N = 20 (14M, 6F; aged 25–29; mean age 27 ± 4,2), Control group N = 20 (15M, 5F; aged 25–39; mean age 28 ± 6,8).	N = 34 Experimental group N = 17 (mean age 29 ± 7), Control group N = 17 (mean age 27 ± 7).	N = 23 (13M and 10F; aged 18–50). Experimental group N = 11 (6M, 5F; mean age 30 ± 10); Control group N = 12 (7M, 5F; mean age 29 ± 9)
ACLR method	Patellar tendon graft	hamstring tendon autograft	hamstring or patellar tendon autograft
Enrollment time	2nd week postoperatively	1 month postoperatively	Individually; within 1st week postoperatively
Intervention	8 weeks of WBV protocol training vs. 8 weeks standard rehabilitation program	4 weeks of WBV training plus standard 4-week rehabilitation program vs. standard 4-week rehabilitation program	24 LVT sessions plus 24 sessions of standard rehabilitation program (ca. 10 weeks) vs. 24 sessions of standard rehabilitation program (ca. 10 weeks).
Outcome measures	At 2nd (baseline), 5th, 8th and 11th week postoperatively: active ROM (goniometer), isometric and isokinetic muscle strength of knee flexors and extensors (dynamometer), postural control (stability platform), knee function (Lysholm Score) Immediately postoperatively and at 11th week postoperatively: knee joint laxity (arthrometer)	At 1st (baseline), 2nd and 3rd month postoperatively: isometric strength of knee flexors and extensors (limb symmetry index; dynamometer); balance (stability platform); knee function (Lysholm Score).	Assessment time was individual for each participant (1st assessment on enrollment, within 1st week; 2nd after last session; ca. at 11th week): knee extensors isometric strength and limb symmetry (force transducer); functional performance (TUG test and 6MWT).
Main outcomes	Significant improvement in experimental vs. control group in postural control (at week 8: 3.3 ± 1.5 vs. 4.9 ± 2.4 (*p* = 0.02); at week 11: 3.1 ± 1.3 vs. 4.7 ± 2.8 (*p* = 0.01)). No difference in experimental vs. control group in ROM, knee laxity, muscle strength or knee function (*p* > 0.05).	Significant improvement in experimental vs. control group in strength symmetry of the knee flexors at 2nd (mean 66% ± 15 vs. 58% ± 13; *p* < 0.05) and 3rd month (77% ± 15 vs. 64% ± 15; *p* < 0.05); knee function at 2nd (mean 11.5 ± 2.8 vs. 7.0 ± 2.7; *p* < 0.001) and 3rd month (23.7 ± 3.4 vs. 16.3 ± 3.1; *p* < 0.001). No difference in experimental vs. control group in strength symmetry of the knee extensors and balance (*p* > 0.05).	Significant difference in experimental vs. control group in PRE–POST extensor muscle strength changes (lower for LVT group; *p* = 0.0049). No difference in experimental vs. control group in limb strength symmetry (*p* < 0.05) and functional tests (*p* < 0.05).

**Table 9:** Summary of reviewed studies on miscellaneous interventions.

Author	Velázquez-Saornil et al. (2017) [36] RCT	Ogrodzka-Ciechanowicz et al. I (2021) [37] RCT	Ogrodzka-Ciechanowicz et al. II (2021) [38] RCT	Clausen et al. (2020) [39] RCT (Pilot Study)
Method of randomization	Opaque closed letter envelopes	Sealed envelopes method	Coin toss	Computer-based randomization
Study population	N = 44 (28M, 16F); Experimental group N = 22 (16M, 6F; aged 19–46; mean age 31.4 ± 8.3); Control group N = 22 (12M, 10 F; aged 19–51; mean age 34.4 ± 8.6).	N = 35 (35M; aged 21–50); Experimental group N = 17 (17M; mean age 30 ± 7.35); Control group (N = 18 (18M; mean age 30 ± 10.42)	N = 38 (28M, 10F, aged 18–40); Experimental group N = 19 (15M, 4F; aged 18–40; mean age 28.2 ± 8.1), Control group N = 19 (13M, 6F; aged 19–39; mean age 27.4 ± 7.8).	N = 26 (12M, 14F; mean age 25.19 ± 8.2) Experimental group N = 14 (6M, 8F; mean age 24.86 ± 9.71); Control group N = 12 (6M, 6F; mean age 25.58 ± 6.4)
ACLR method	Patellar or hamstring tendon grafts	Hamstring tendon graft	Semitendinosus tendon autograft;	Hamstring graft
Enrollment time	12–19 days postoperatively	11 days postoperatively	1st day postoperatively	Immediately postoperatively
Intervention	one-time vastus medialis trigger point dry needling plus 5 weeks of rehabilitation program vs. 5 weeks of rehabilitation program	6 months of High Tone Power Therapy duration 1h, after every rehabilitation session) plus rehabilitation program vs. 6 months of rehabilitation program	10 days of magnetic field (each day, 30′) plus postoperative instructions vs. 10 days of placebo magnetic field (each day, 30′) plus postoperative instructions	3 weeks of app-based serious gaming (5 times daily) plus rehabilitation program vs. rehabilitation program
Outcome measures	At baseline, immediately after intervention, 24 hours, 1 and 5 weeks after the first TrPDN intervention: Pain intensity (VAS), ROM (goniometer), and function (WOMAC).	2 days before and 6 months after surgery: muscle strength (dynamometer), ROM (goniometer), knee and thigh circumference (tailor’s tape), knee function (Lysholm test) and pain (VAS).	At baseline, each time after therapy for 11 days: knee effusion (circumference; tape measure). At 1st and 11th day: AROM (goniometer).	Before operation and after 6 weeks postoperatively: knee function (IKDC, Lysholm Knee Score, Tegner scale, KOOS), maximum strength, pain (VAS), and knee circumferences (10 cm and 20 cm above knee)
Main outcomes	Significant difference in experimental vs. control group (*p* < 0.001) in pain (increased in TrPDN; after intervention 6.86 ± 0.9 vs. 6.57 ± 0.9), ROM (at 1st: 98.57 ± 8.5 vs. 89.52 ± 8.6; 2nd: 104.76 ± 9.8 vs. 90 ± 9.5; and 3rd measurement: 131.43 ± 11.5 vs. 113.81 ± 11.6), and knee function (at 2nd: 64.48 ± 3.7 vs. 67.75 ± 4.2; 3rd: 38.51 ± 9 vs. 46.18 ± 10.9; and 4th measurement: 14.08 ± 3.7 vs. 18.6 ± 3.9).	Significant improvement in experimental vs. control group in extensors strength (23.28 ± 0.7 vs. 20.19 ± 0.6; *p* = 0.028), knee (35.00 ± 1.80 vs. 38.00 ± 2.18; *p* = 0.043) and thigh circumference (46.00 ± 3.86 vs. 41.00 ± 3.11; *p* = 0.033), knee extension (0.00 ± 0.15 vs. 3.00 ± 0.99; *p* = 0.048), and function (94 ± 7.01 vs. 85 ± 8.71; *p* = 0.035). No difference in experimental vs. control group in pain (*p* > 0.05).	No difference in experimental vs. control group in knee joint effusion and AROM (*p* > 0.05).	Significant improvement in experimental vs. control group in relative change in maximum strength (1.7 ± 1.17 vs. 1 ± 0.13; *p* = 0.03). No significant changes in knee function and pain (*p* > 0.05).

## Data Availability

The datasets used during the study are available from the corresponding author on reasonable request.

## Appendix A

**Table A1:** Databases search equations.

PUBMED/MEDLINE: (ACL OR (anterior cruciate ligament) OR reconstruction OR (injury AND (repair OR surgery))) AND (rehabilitation OR physiotherapy OR (physical therapy) OR training OR exercise OR intervention OR treatment OR (standard rehabilitation program)) AND ((early phase) OR (acute phase) OR (initial phase)) AND (pain OR edema OR swelling OR (range of motion) OR ROM OR (muscle strength) OR (knee function) OR (knee activity)) WEB OF SCIENCE: (ACL OR (anterior cruciate ligament) OR reconstruction OR (injury AND (repair OR surgery))) AND (rehabilitation OR physiotherapy OR (physical therapy) OR training OR exercise OR intervention OR treatment OR (standard rehabilitation program)) AND ((early phase) OR (acute phase) OR (initial phase)) AND (pain OR edema OR swelling OR (range of motion) OR ROM OR (muscle strength) OR (knee function) OR (knee activity)) SCOPUS: (ACL OR (anterior cruciate ligament) OR reconstruction OR (injury AND (repair OR surgery))) AND (rehabilitation OR physiotherapy OR (physical therapy) OR training OR exercise OR intervention OR treatment OR (standard rehabilitation program)) AND ((early phase) OR (acute phase) OR (initial phase)) AND (pain OR edema OR swelling OR (range of motion) OR ROM OR (muscle strength) OR (knee function) OR (knee activity)) SCIENCE DIRECT: (ACL reconstruction) AND (rehabilitation OR physiotherapy) AND (early phase OR acute phase) AND (pain OR range of motion OR edema OR strength))))
